# Are Multimorbidities Underestimated in Scoring Systems of Stevens-Johnson Syndrome and Toxic Epidermal Necrolysis Like in SCORTEN?

**Published:** 2012-08-02

**Authors:** Tobias von Wild, Peter L. Stollwerck, Thomas Namdar, Felix H. Stang, Peter Mailänder, Frank Siemers

**Affiliations:** Department of Plastic and Hand Surgery, Burn Unit, University Hospital Schleswig-Holstein Campus Lübeck, Ratzeburger Allee 160, 23538 Lübeck, Germany

## Abstract

**Objective:** Toxic epidermal necrolysis and Stevens-Johnson syndrome have related high morbidity and mortality. We predict that preexisting multimorbidity is a major prognostic factor of both these diseases. **Methods:** A retrospective analysis in toxic epidermal necrolysis and Stevens-Johnson syndrome patients over the past 10 years. Three severity categories (minor, moderate, and severe multimorbidity) were defined according to a point-rating system. **Results:** Twenty-seven inpatients, with a median age of 63 years, diagnosed with toxic epidermal necrolysis (n = 13) or Stevens-Johnson syndrome/toxic epidermal necrolysis (n = 14) were assessed in this study. Of these, 14 patients died during the course of the study. Nonsurvivors showed significantly higher multimorbidity (*P* = .038), with higher scoring on the points system for disease severity (*P* = .003), than survivors and CART (Classification and Regression Trees) cross-validation (*P* < .05). **Limitations:** Restricted number of patients due to low prevalence rate. **Conclusion:** The complexity of associated multimorbidity appears to have a large influence on toxic epidermal necrolysis and Stevens-Johnson syndrome prognosis, which has not been considered in any of the established scoring systems.

Toxic epidermal necrolysis (TEN) and Stevens-Johnson syndrome (SJS) are rare diseases with a prevalence of 1 to 2 new cases per million each year. Both diseases are associated with high morbidity and a mortality rate of approximately 85%. There is a high risk of super-infection and sepsis due to subepidermal and mucosal blisters, like superficial second-degree burn wounds. In addition to the initial diagnosis of SJS/TEN, patients often present with multiple morbidities (MMs), which may be important predictors of outcome. The use of multimorbidity is becoming increasingly more common to describe the co-occurrence of multiple chronic or acute diseases in a single patient, without reference to an index condition,^1^^,^^2^ whereas comorbidity is the presence of additional (chronic) diseases in relation to the index disease. As chronic and acute diseases unrelated to the index disease are considered herein, we used the expression of multimorbidity to describe the conditions. Currently, there is a great need for a point-scoring system to determine potential predictors of prognosis and mortality of SJS and TEN patients. Furthermore, the number and severity of serious MMs have not been included as prognostic factors in these scoring systems. The most common used predictor SCORTEN (TEN scoring system), a validation system of 7 criteria, only includes malignancy.

The study described herein was based on a 10-year retrospective analysis of the major influences of MM on prognosis.

## MATERIALS AND METHODS

Twenty-seven patients with highly confirmed clinical and histopathological diagnosis of TEN or SJS were treated over a 10-year period. A clinical diagnosis was defined by the validated results of the Documentation Center for Severe Skin Reactions of the German Registry, Freiburg, which induced the leading RegiSCAR study. All patient data were analyzed retrospective to age, sex, necessity of ventilation, catecholamine administration, occurrence of skin reaction inside or outside the hospital, and outcome.

Each individual prognosis was also calculated by the widely used TEN scoring system, SCORTEN, if sufficient data were available. Each of the 7 criteria receiving 1 point were as follows: (1) age more than 40 years, (2) malignancy, (3) tachycardia more than 120 per minute, (4) blistering more than 10% of total affected body surface area (TBSA), (5) urea serum more than 10 mmol/L, (6) bicarbonate less than 20 mmol/L, and (7) glucose serum more than 250 mg/dL.

As multimorbidity has still not been considered in the scoring, we analyzed its impact on the prognosis of TEN/SJS patients. To this end, we decided to define 3 severity categories with a point rating system: (1) minor MMs—have no or trivial impact on outcome during hospitalization; (2) moderate MMs—have classical risk factors defined by the World Health Organisation; and (3) severe MMs—have life-threatening or life-shortening morbidities, increasing the likelihood of poor outcome with or without treatment. Postoperative immunodeficiency, malignancy, AIDS, and embolism are examples of MMs that are associated with TEN/SJS. As an assessment of the TBSA is prone to a subjective judgement of redness and bullae, this criterion was excluded.

Each potential risk was correlated with information obtained through guideline research (AWMF, PubMed, Medline). The 3 categories mentioned earlier were chosen, as we believe that MMs should be rated according to their severity. Hence, mild, moderate, or severe MMs receive 1, 2, or 3 points, respectively. Each patient was analyzed individually by the number and severity of MMs, and the risk was calculated by their total point scoring. For better illustration, all included diseases and their assignment are shown in Tables 1 and 2. These lists do not claim completeness of all existing morbidities rather than presenting morbidities of our patient's cohort. Multivariate analyses were performed by the CART (Classification and Regression Trees) method.^3^^,^^4^ As predictive variables for mortality, the following parameters were included into the model: age, overall points of our new scoring system, gender, and the number of systemic cardiac diseases, in respect to necessity of fluid administration. SPSS Expert 15.0 software was used for statistical analyses.

## RESULTS

### Demographic characteristics

TEN (n = 13), SJS (n = 1), or SJS/TEN overlap (n = 13) were confirmed histologically in 27 patients, as shown by the patient series in Table 1. The 18 female and 9 male patients were aged 21 to 88 years [median 63, interquartile range (IQR) 29].

With regard to age and outcome, survivors had a median of 59 years (IQR 6), whereas nonsurvivors had a median age of 67 years (IQR 33). Although the range of median age varied considerably, the IQR of the nonsurvivors showed a larger range than the survivors, as there was also morbidity among younger patients. A significant age-dependent mortality was not seen by the Mann-Whitney test for independent factors (*P* = 0.197).

When subdivided into gender and subgroups of SJS/TEN versus TEN, the distribution was almost equal in the SJS/TEN overlap (8 female and 6 male patients), of which 5 female and 1 male patients died. Ten female and 3 male patients presented with a diagnosis of TEN, of which 6 female and 2 male patients died. There was no statistically significant difference in mortality between sexes (Fisher exact test: *P* = 0.163), although the small number of each group could be a limitation in this result.

Formation of bullae was seen in 13 patients during outpatient treatment and 14 patients during hospitalization, with 6 cases occurring postoperatively. Detailed information about mucosal eruption was incomplete.

The time lapse between eventual first manifestation and admission to our intensive care ward was 0 to 39 days (median 2, IQR 6). No statistical significance could be seen in mortality by the Mann-Whitney *U* test (*P* = 0.116).

### SCORTEN—Results

No patients scored 1 point or less, with a survival prediction of 100%. In total, mortality was predicted as 35.3% or 77.8% in 10 patients (SCORTEN 4 and 5 points), of whom 4 survived, with 3 scoring 5 points. In contrast, mortality prediction of 13 patients was as low as 6.7% and 23.8% (SCORTEN 2 to 3 points), of whom approximately 50% died. In conclusion, prediction based on SCORTEN was accurate for only 55.56% patients.

### Multimorbidity

Our patient series showed a median of 4 MMs per patient (0-8 MMs) (Table 1), with 63 different MMs being diagnosed, as shown in Table 2.

On the basis of the categorization as described earlier, our data showed on average 1.7 mild (median 1), 3.1 moderate (median 3), and 2.3 severe (median 2) MMs per person.

When divided into 2 subgroups based on patient mortality, our data revealed that nonsurvivors had 2 to 8 MMs (median 4.5 MMs, IQR 1.75; 0 mild MM, IQR 1; 2 moderate MMs, IQR 3.5; and 1.5 severe MMs, IQR 2.5; 11.5 points overall score, IQR 8.25).

During our study, 11 out of 14 nonsurvivors had 1 to 5 life-shortening MMs, 10 of whom had another moderate MM. Three nonsurvivors without any severe MM were aged between 77 and 88 years and suffered from 1 to 6 moderate MMs. Only one of the 17 survivors had a severe MM, with malignant media infarction. The survival group showed 0 to 6 MMs (median 3.0 MMs, IQR 3.5; 1 minor MM, IQR 2; 1 moderate MM, IQR 3; and 0 severe MM, IQR 0; 4 points overall score, IQR 6.5). One patient, without any chronic disease, had an infection of the urinary tract and received antibiotic trigger medication. Four surviving patients incurred minimal MMs, such as flue, conjunctivitis, or organic brain syndrome.

The majority of MMs in the survival group were well-known risk factors, such as systemic cardiac (arterial hypertonia and coronary heart disease), kidney and lung diseases, diabetes mellitus, and rheumatoid disorders.

**Figure 1:**
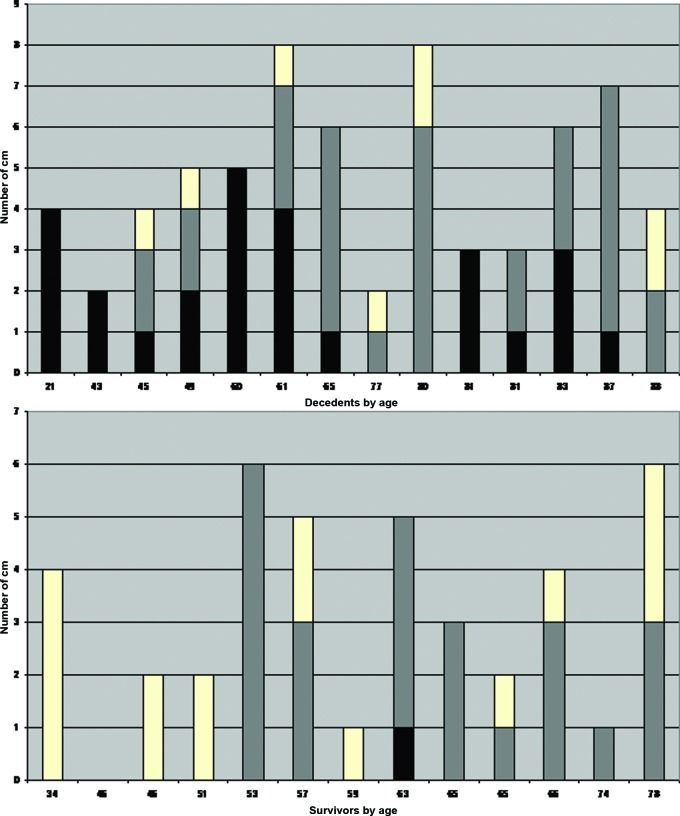
Column diagram with number of MMs of nonsurvivors and survivors by age in years. (white: mild MMs; grey: moderate MMs; black: severe MMs).

Figure 1 shows the diversity in the number and severity of MMs of nonsurvivors and survivors according to age. Severe MMs (in black) are more common per patient in the nonsurvivor group, while mild MMs (in white) appear in the survivor group. Moderate MMs (in grey) are almost equal between both groups.

**Figure 2:**
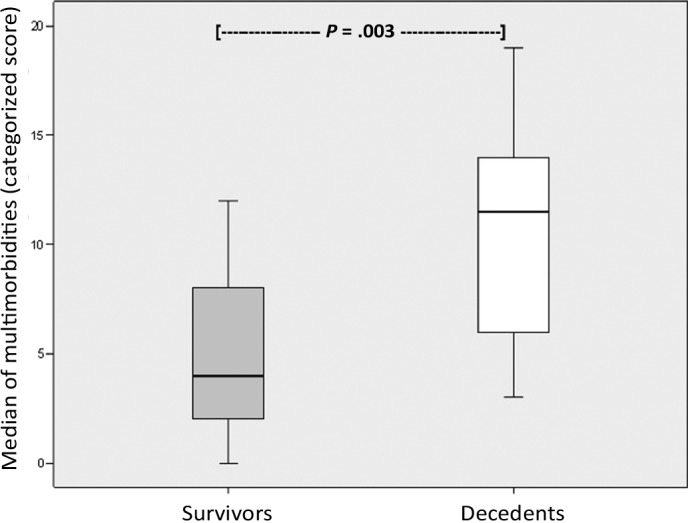
Influence of median overall score on outcome. Box blot showing a high significance of median overall score of the individual subgroups of MMs between survivors and nonsurvivors.

**Figure 3:**
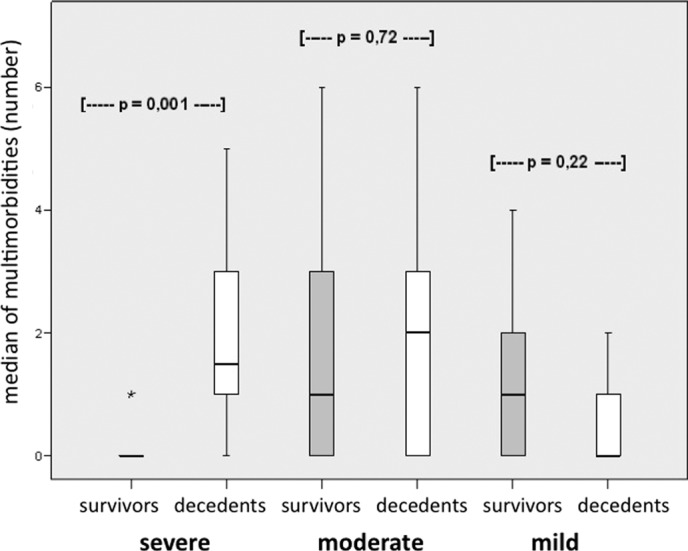
Influence of MM subgroups on outcome. Box blot showing the influence of MM subgroups on outcome. Patients with severe MMs have a higher mortality rate; mild MMS have a higher survival rate; and no trend seen in moderate MMs.

Using the Mann-Whitney *U* test for statistical analysis of independent variables, a significant difference could be seen in the number of MMs in survivors compared with nonsurvivors, with a higher incidence in nonsurvivors (*P* = 0.038). A highly significant difference could also be seen in the total score for each MM subgroup according to our scoring system (*P* = 0.003) (Fig 2). In the respective groups of survivors and nonsurvivors, patients had a very similar incidence of moderate MMs (IQR 3 vs 3.5). Survivors had a higher number of minimal MMs, whereas nonsurvivors had high incidence of severe MMs (*P* = 0.001) (Fig 3). As a result of these multivariate analyses of CART, the patient cohort (n = 27) was divided into 2 groups (= end nodes) according to total score following our new scoring system. The first subgroup, which was defined by 12 or less points (= cutoff point), included 21 patients, 8 of whom died (38%). All patients (n = 6) in the second group, with more than 12 points, died (100%). Cross-validation shows *P* < 0.05. In addition, the importance of age in different classification analyses was outlined by an alternative cutoff point of 77 years of age, which indicates that, in general, older patients suffer from more severe morbidities.

Concerning our question of whether preexisting cardiac diseases influence patient outcome, Fisher exact test had a *P* value of 0.013, with the number of MMs also being significant (*P* = 0.038, Mann-Whitney test).

Catecholamine application was necessary in 12 cases, of which 11 died. Chi-square test revealed a high significance between the requirement of catecholamines and mortality (*P* ≤ 0.0001; odds ratio 44; confidence interval: 3.966-488.188). With regard to the 2 groups defined by the CART analyses, all patients with more than 12 points total score, and who also died, required catecholamine application. A correlation between necessity for catecholamines and the total points of MM assigned by our categorization system was significant (Mann-Whitney *U* test, *P* = 0.028). However, only a tendency toward the total number of MMs could be reached (Mann-Whitney *U* test, *P* = 0.067). Statistical analysis showed a significant difference between the requirement for catecholamine application and subdivision of histopathological diagnoses (TEN: *P* = .008, odds ratio 4.0; confidence interval: 1.205-13.283; SJS/TEN: *P* = 0.013, odds ratio 30.1; confidence interval: 471- 611.797).

## DISCUSSION

The incidence of TEN or SJS is low, with only 1 to 2 new cases per million population each year^5^; however, mortality in these patients reaches approximately 85%.^6^^-^13 TEN and SJS are histopathologically identical, but differ in the dimension of affected body surface area.^14^^,^^15^ Owing to subepidermal blisters, such as superficial second degree burn wounds, there is a high risk of superinfection and sepsis. Apart from the extent of epidermolysis, the existence of multimorbidity appears to have the most significant effect on prognosis. Nevertheless, MMS are not included in established scoring systems, such as the SCORTEN.

The likelihood of taking medication that could potentially trigger MMs rises with increasing age, along with the chance of sustaining SJS and TEN. In the current literature, approximately 75% of patients with SJS/TEN are older than 40 years (mean age 54.4 years; range 1-94 years)^6^^,^^8^^,^^16^^,^^17^; However, these studies do not consider the influence of preexisting MMs on outcome of the disease.

There is a great need for a scoring system that incorporates potential predictors for prognosis and mortality of SJS and TEN.^6^^,^^8^^,^^16^^,^^17^

Bastuji-Garin et al developed a specific TEN scoring system (SCORTEN) that makes inter-individual prognoses in SJS/TEN patients^18^^-^19 based on seven criteria described earlier, each of which receives one point. If the SCORTEN was 0-1 point, mortality is 0% (95% confidence interval, 0-8.5); 6.7% (1.4–18.3) with 2 points; 23.8% (8.2–47.2) with 3 points; 35.3% (14.2–61.7) with 4 points; and 77.8% (52.4–93.6) with 5 or more points. Furthermore, the most accurate prognosis in Bastuji-Garin studies could only be made on day three, as the score was too low on the days previous and too high the days after. This scoring system is confirmed in other studies, and is considered to be the gold standard for prognosis.^20^^-^25 However, we believe that the SCORTEN scoring system should be complemented by further aspects as there is a discrepancy between prognosis by SCORTEN and actual outcome, with an accuracy of 55.56%. The highest discriminatory power of SCORTEN seems to be influenced by the day of evaluation.^18^^,^^19^ The time period lapsing between the first manifestation and admission to the intensive care ward differs dramatically, as can be seen by our data (0–39 days).

In our opinion, variations in biological tests (heart rate, hyperglycemia, elevated urea, and decreased bicarbonate) are affected by parameters such as time of blood sampling, state of vigilance, and pain during measurements. The 2 SCORTEN parameters incorporating age more than 40 years and TBSA (>10%), with or without inflicted mucosa, are very likely predictors, as can be seen in our cohort. Determining the affected TBSA requires experience in the examination and is still subjective, often leading to overestimations.

Furthermore, other groups are also critical of the SCORTEN method and believe that further assessments are needed for it to be more accurate.^26^^-^29 Vaishampayan et al^29^ suggest a reweighting of included parameters and the inclusion of preexisting diseases. Spornraft-Ragaller et al^28^ believe that severe cases are not differentiated sufficiently, whereas Hague et~al^26^ find the respiratory involvement is not reflected highly enough. Sekula et al^27^ defined an auxiliary score using the great amount of data obtained in the RegiSCAR study and the earlier EuroSCAR study. They showed a statistical improvement in predictive ability using other clinical criteria compared with SCORTEN. In our opinion, chronic morbidities other than malignancies have to be critically discussed to have an influence on outcome in TEN/SJS for reasons described earlier. Preexisting well-established multimorbidity rating systems, such as the Charlson, Kaplan, Angold and Elixhauser indices, are in use, but they do not appear to be useful in these types of diseases.^1^^,^^30^^-^34 First, it is difficult to define a term for the coexistence of morbidities with the initial disease, including complications. Terms, such as multimorbidity, comorbidity, burden of disease, and frailty, are often used interchangeably. Second, most studies have examined comorbidities on limited sets of patients and have only been developed for disease with long-term follow-up periods, such as breast cancer or nephropathies.^34^^-^47 However, medication-associated serious exfoliative skin diseases, like TEN or SJS, are acute disease, which heal fully if survived. Thus, there is a short time period for survival, with a large requirement for medication and a high incidence of one or more other morbidities. Elixhauser et al^32^ described comorbidities as having independent effects on outcomes and stated that they should not be simplified in an index as they can affect outcomes differently among different patient groups.

In our opinion, chronic morbidities other than malignancies have to be critically discussed as having an influence on the outcome in TEN/SJS, for reasons described herein. On the basis of our new categorization system of multimorbidity subgroups, which considers their total number and intraindividual severity, we could significantly calculate patient prognosis.

As expected, patients with a high number of severe MMs, which singularly harbor a higher risk of mortality, had a higher mortality rate than patients with moderate or mild MMs. None of the patients died with no or mild MM, even if they had to be ventilated for up to 52 days.

Because of the low incidence of medication-associated serious exfoliative skin diseases, it is difficult to recruit an adequate number of patients to be representative. As shown by the Center of Quality and Management in Health Care, Medical Council Niedersachsen, the number of cases in our study is sufficient to show the impact of MM on the prognosis of medication-associated serious exfoliative skin diseases. However, a multicenter study may be more effective at obtaining a larger number of patients and more significant results.

## CONCLUSION

Current literature does not consider the complexity of preexisting MMs in the prognosis of TEN/SJS, even though they seem to have a great impact on patient outcome. We do not suggest that our scoring system should replace other well-established and well-tested existing morbidity scoring systems, but we do believe that multimorbidity, which appears to have a significant effect on outcome from our results, should be included in these scoring systems. Although the number of patients in our group was quite small, the influence of MM on the prognosis of clinical outcome was to be statistically significant.

## Figures and Tables

**Table 1:** Patients' characteristics

Case	Sex	Age	Diagnose	TBSA (%)	Katecholamins	Number of MM	Mild MM	Moderate MM	Severe MM	Overall score	SCORTEN	Outcome
1	f	21	TEN	95	+	4	0	0	4	12	2	†
2	m	34	SJS/TEN	24		4	4	0	0	4	2	
3	m	43	SJS/TEN	18		2	0	0	2	6	3	†
4	f	45	TEN	80	+	8	1	2	1	8	4	†
5	f	46	TEN	20		0	0	0	0	0	2	
6	f	46	SJS/TEN	10	+	2	2	0	0	2	2	
7	m	49	TEN	40		5	1	2	2	11	5	†
8	m	51	SJS	6		2	2	0	0	2	2	
9	f	53	SJS/TEN	14		6	0	6	0	12	2	
10	f	57	TEN	31		5	2	3	0	8	2	
11	m	59	SJS/TEN	11		1	1	0	0	1	5	
12	f	60	SJS/TEN	28	+	5	0	0	5	15	5	†
13	f	61	TEN	35	+	7	0	5	2	16	4	†
14	f	63	SJS/TEN	15		5	0	4	1	11	3	
15	m	65	SJS/TEN	45		3	0	3	0	6	3	
16	m	65	TEN	55	+	6	0	5	1	13	3	†
17	f	65	TEN	45		2	1	1	0	3	5	
18	m	66	SJS/TEN	12		4	1	3	0	7	5	
19	f	74	TEN	35	+	1	0	1	0	2	2	
20	f	77	TEN	90		2	1	1	0	3	3	†
21	m	78	TEN	65		6	3	3	0	9	4	
22	f	80	SJS/TEN	24	+	8	2	6	0	14	2	†
23	f	81	TEN	35		3	0	0	3	9	4	†
24	f	81	SJS/TEN	25	+	7	0	2	1	7	5	†
25	f	83	SJS/TEN	20	+	6	0	3	3	15	2	†
26	f	87	SJS/TEN	11	+	25	0	6	1	15	3	†
27	f	88	TEN	40	+	61	2	2	0	6	3	†

+ Patients have received katecholamin.

† Patients have died.

TBSA indicates body surface area; MM, multimorbidity; SJS, Stevens-Johnson syndrome; TEN, toxic epidermal necrolysis.

**Table 2:** Preexisting morbidities of our collective

Mild Diseases 1 Rating Point	Moderate Diseases 2 Rating Points	Severe Diseases 3 Rating Points
Conjunctivitis	Adipositas per magna	AIDS
Cough	Alcohol abuse	Adult respiratory distress syndrome
Coxarthrosis	Aortic aneurysm	Disseminated intravascular coagulopathy
Epilepsy	Arrhythmia absoluta	Recurrent embolia
Gout	Arterial hypertonia	Incarcerated perforated hernia of small bowel
Lupus erythematosus	Arteriosclerosis	Malignant media infarction
Mental retardation	Asthma bronchialis	Mass transfusion due to consumption coagulopathia
Normal pressure hydrocephalus	Cardiac insufficiency	Necrotic bronchopneumonia
Organic brain syndrome	Cerebellum infarction	Osteosarcoma with granulocytopenia
Psoriasis	Compensated kidney insufficiency	Pneumothorax
Rheumatoid arthritis	Chronic obstructive pulmonary disease	Post operative hemorrhage post total endoprosthesis
Sec. parathyroidism	Coronary heart disease	Postoperative complication immunosuppression
Trigeminal neuralgia	Diabetes mellitus type II	Recurrent lung embolia
Urinary tract infection	Endocarditis	Rhabdomyolysis
	Hepatic cirrhosis	Small bowel perforation
	Mitral valve replacement	Wertheim surgery with intestinal perforation
	Nephropathy	
	Pancreatitis	
	Pneumonia post heart infarction	
	Respiratory partial insufficiency	
	Thromboses	
	Toxic hepatic fibrosis	
	Zoster-meningoencephalitis	
